# “Smashing through barriers”? A multimodal critical discourse analysis of media representations of hearing loss and D/deafness

**DOI:** 10.1371/journal.pone.0342462

**Published:** 2026-02-13

**Authors:** Sophie Fawcett-Jones, Emma Putland, Sian Calvert, Emma Broome, Helen Henshaw, Tom Dening, Clare Burgon, Eithne Heffernan

**Affiliations:** 1 School of Medicine, University of Nottingham, Nottingham, United Kingdom; 2 Department of Linguistics and English Language, Lancaster University, Lancaster, United Kingdom; 3 Hearing Sciences, Mental Health and Clinical Neurosciences, University of Nottingham, School of Medicine, Nottingham, United Kingdom; 4 National Institute for Health and Care Research (NIHR) Nottingham Biomedical Research Centre, Nottingham, United Kingdom; 5 School of Sport, Exercise and Health Sciences, Loughborough University, Loughborough, United Kingdom; Southwest University, CHINA

## Abstract

Hearing loss (HL) is a prevalent condition that can substantially impact quality of life. Hearing aids can benefit people living with HL, yet many delay seeking treatment. This may be due to limited public awareness of HL and the stigma surrounding HL and hearing aids. The media can significantly shape public perceptions of HL and D/deafness, and there have been calls for improved media portrayals of HL and D/deafness. This study examined how British newspapers represent HL and D/deafness both visually and textually, and whether these representations reiterate and/or challenge stigma. This qualitative study used multimodal critical discourse analysis (MCDA) guided by stigma theory and the Visual Discourses of Disability framework. Public contributors living with HL or D/deafness were consulted. A Nexis database search retrieved 7,173 articles about HL or D/deafness from 2022–2023. A random sample, extracted from the 200 most relevant articles, was screened. Three key themes were identified: (1) representing social progress, including technological advancements and societal roles for people with HL or D/deaf people, (2) the lack of diverse narratives and perspectives, including the absence of older adults, and (3) the stigma and social barriers associated with living in a hearing-orientated world, including tensions regarding whether HL should be (in)visible. Combined, this suggests that the overall social progress narrative is challenged by continued stigmatisation and inadequate diversity. This research was a novel application of MCDA to representations of HL and D/deafness, which focused on British newspapers. Further efforts are needed to improve these representations, particularly representations of older adults. Future research should apply MCDA to representations of HL and D/deafness in other contexts. The findings have important implications for academics in discourse and disability studies, and for all those who communicate with the public about hearing loss, including researchers, clinicians, public health officials, charities, and the media.

## Introduction

Hearing Loss (HL) affects nearly 20 per cent of the global population, or 1.5 billion people [1]. Within the United Kingdom (UK), as many as one in three adults, or 18 million people, experience HL [2]. In addition, approximately 87,000 people are members of the UK Deaf community [3]. HL is an age-related condition and thus is especially common amongst older adults, with 80 per cent of those aged over 70 years having HL [4]. This long-term condition, particularly when left untreated, can place a severe burden on individuals by significantly impairing their communication, social and occupational functioning, mental well-being and overall quality of life [5]. Studies demonstrate that people with HL are at greater risk of developing mental health conditions [6,7]. Notably, HL is associated with depression, anxiety, psychological distress and increased use of mental health services [8,9]. Additionally, HL can shrink individuals’ social networks (i.e., objective social isolation) and place them at a higher risk of loneliness (i.e., subjective social isolation) [10,11]. There is also evidence of a link between cognitive impairment and HL. The Lancet Commission on dementia identified HL in mid-life onwards as a potentially modifiable risk factor for dementia [12].

There are many aural rehabilitation interventions available to help people to manage their HL, including hearing aids, cochlear implants, and programmes to improve communication and speech-reading [13]. Aural rehabilitation interventions can improve quality of life and social participation and decrease the risk of psychological distress [8,14,15]. However, many people live with HL for several years before receiving appropriate care and there are suboptimal uptake and adherence rates for aural rehabilitation interventions [16]. For example, a study from the United States of America (USA) found that 75 per cent of HL cases go under-diagnosed and under-treated [17]. An analysis of data from the English Longitudinal Study of Ageing found that almost 40 per cent of those who acknowledged having HL did not report it to their primary care provider. Of those who did report it, many were not referred to secondary care (e.g., audiology services, ENT) despite clinical guidelines advising primary care professionals to refer anyone suspected of HL for further assessment [16]. Amongst people living with HL who do attend audiology services, many do not opt to obtain hearing aids or do not use their hearing aids regularly [18,19]. Common reasons for this include difficulties maintaining hearing aids, discomfort, and perceived lack of benefit [19,20]. There is also a stigma surrounding HL and hearing aids, including associations with ageing and disability, that can lead people to hide their HL and to reject hearing aids, as they are a visible signifier of HL [21]. Furthermore, research has shown that many members of the public and health professionals have limited knowledge about HL and do not regard hearing healthcare as a priority [22,23].

The news media have the power to shape ideas and behaviours relating to health conditions [24,25]. Indeed, newspapers are ‘still considered the most important medium through which to understand the world’ [26]. However, the primary goal of the media is to engage their audience, rather than to challenge misconceptions and stigmas surrounding health conditions [26]. The media can also reinforce the stigmatisation of health conditions. For instance, advertisements for hearing aids in the media were previously found to highlight their easy concealment, suggesting HL should be hidden [21]. Therefore, it is crucial to study how HL is represented in the media and to consider how these representations might contribute to the stigma surrounding HL.

Research has begun to examine the media’s representation of people living with HL, deaf people and/or people from the Deaf community in different countries. The term HL is commonly used to describe individuals with varying degrees of HL, ranging from mild to severe. The term ‘deaf’ (with a lower case ‘d’) typically refers to people with profound HL that is not associated with their cultural identity. This differs from the Deaf community (with an upper case ‘D’), which has its own vibrant and unique culture and whose members often use sign language as their primary language [1,27,28]. Studies from the USA examined representations of people living with HL, deaf people or people from the Deaf community on television and in newspapers. They found that the frequency of stories and characters that related to these groups has increased over time and that more recent portrayals of these groups and hearing aids are more realistic than previously shown [29,30]. For example, Foss [29] found that some television characters experienced embarrassment, denial and isolation after they identified their HL, which is similar to the experiences reported by many people in real-life [10]. However, the depiction of television characters from these groups in the USA appears to be limited regarding the ages, genders and races shown [29]. Some studies found that most portrayals feature older adults and men, whereas other studies found that mostly young Caucasian women are featured [29,31].

Manzi et al. [32] analysed the linguistic expressions within media representations of hearing aids in Italy, finding that the language used was formal, technical and medically oriented. Viewers reported via online surveys that this negatively impacted their attitudes towards hearing aids. The authors proposed that such representations can promote illness-related stigma and discourage hearing aid uptake. However, Koerber et al. [33] thematically analysed the representation of workers with HL in Canadian newspapers and found that they were praised for being creative problem-solvers who found ways to work despite having HL. Rather than normalising success in people with HL, some articles described individuals with HL as heroes for overcoming the challenges of HL and striving towards a sense of ‘normality’ [33]. This could be viewed as an example of the ‘supercrip’ narrative, whereby people with disabilities are represented within the media as an inspiration and are raised to hero status for overcoming the consequences of their disabilities [34,35]. This narrative can foster the unrealistic expectation that people can prevail over the effects of their disability through perseverance alone, with insufficient consideration for the social barriers that they face [35,36]. This narrative is dependent on an ableist view that places low standards on people with disabilities and assumes they have inferior abilities [35,36].

Building upon the existing literature and calls to further examine and improve the news media’s representation of HL and D/deafness [30,37,38], this research aims to examine how newspapers represent HL and D/deafness in a UK context. This will encompass representations of people living with HL, deaf people and members of the Deaf community. We have used the terms ‘HL’ and’D/deafness’ alongside ‘people living with HL’ and’D/deaf people’ throughout this paper to recognise the diversity within the population, and the different audiological and cultural experiences associated with HL and D/deafness. Specifically, this paper asks:

1. How do contemporary UK newspapers represent HL and D/deafness, as well as people living with HL and D/deaf people?
2. How might these representations reiterate and/or challenge existing stereotypes and stigma surrounding HL and D/deafness?

To the authors’ knowledge, this is the first study to examine this topic in UK newspapers, which will enable the comparison of UK specific representations of HL with those of other countries. This study uses a qualitative approach, Multimodal Critical Discourse Analysis (MCDA), to gain an in-depth understanding of the language (written representations) and images (visual representations) used to depict HL and D/deafness within UK newspapers. While a new approach for HL and D/deafness, MCDA has been applied to various conditions, including dementia, for which stock images and news reports have been shown to provide misleading and stigmatising depictions [39,40]. This research is informed by stigma theory and takes a social constructionist approach to stigma. It regards stigma as a social process whereby members of marginalised groups are positioned as undesirable according to socially constructed criteria, and are subsequently subject to discrimination [41]. Social discourses are key to this process. This research follows the Foucauldian tradition, defining discourses as (prevalent) ways of representing people, animals, things and events, which not only reflect but help to construct and maintain how they are understood and positioned in social life [42]. We believe that this is the first paper to apply this analytical approach to HL and D/deafness in the media.

## Materials and methods

This research entailed a qualitative review of publicly available newspaper articles and consulting a Patient and Public Involvement (PPI) panel. Therefore, ethical approval was not required. The Standards for Reporting Qualitative Research checklist (S1 File) guided the reporting of this project [43].

### Data selection

A search was conducted by the first author in May 2023 using Nexis, a database of news, business and legal sources, to find published UK newspaper articles that focused on HL. Eligibility criteria for the articles were: (1) written in the English language, (2) primary focus on HL and D/deafness, (3) news stories, not advertisements or letters, (4) about chronic, rather than acute, HL, which could range from mild to profound, (5) about HL and D/deafness in adults only, (6) published in national newspapers, and (7) published between 1^st^ May 2022 and 30^th^ April 2023 only. The timeline of a year was chosen to capture a spread of articles and reduce the impact of one prominent news story. This timeline was also selected because the study focused on recent, rather than historical, media representations.

Newspapers were selected as they are a major source of information about HL and D/deafness for the public and may influence help-seeking [30]. The PPI panel confirmed that they are an important source to examine. Six of the most widely read and circulated newspapers in the UK were searched (see Table 1). They included three tabloids and three broadsheet newspapers to examine a variety of HL portrayals. Generally, tabloids are image led, whereas broadsheets are text led [44].

**Table 1:** Number of articles retrieved, screened and analysed for each newspaper.

Newspaper	Type	Retrieved	Screened	Included in Analysis
**Mail**	Tabloid	2,396	7	2
**The Sun**	Tabloid	1,256	4	3
**Mirror**	Tabloid	1,144	6	5
**The Independent**	Broadsheet	895	10	6
**The Guardian**	Broadsheet	467	2	2
**The Times**	Broadsheet	1,015	3	2
**Total**		**7,173**	**32**	**20 (and 53 images)**

The Nexis database was searched using search terms (S2 File) that were developed by examining the HL and D/deafness literature, particularly systematic reviews [5,14,45]. The broadsheet search retrieved 2,377 articles, whilst the tabloid search retrieved 4,796 articles. In each search, the articles were ordered by relevance within Nexis. The top 100 broadsheet articles and the top 100 tabloid articles in terms of relevance were extracted for screening. Additional articles could be extracted if required.

### Screening

Table 1 shows the total number of articles retrieved, screened, and analysed for each newspaper. To ensure a random selection of articles, every tenth article was examined for the tabloids and broadsheets to determine eligibility. The full text and images within each article were examined. All articles were screened by the first author, and a sample was independently screened by a second researcher. A third researcher was consulted to resolve any disagreements. As this was a qualitative study, there was no pre-determined sample size, although it is comparable to previous studies of dementia representations that analysed 11–31 articles [39,46]. Screening continued until saturation was reached, or the point at which little new information that was salient to the research aims could be identified from additional articles. Saturation was assessed via a preliminary data analysis and discussions at research team meetings. The main reasons that articles were excluded were as follows: main focus not on HL or D/deafness, wrong population (e.g., children), or wrong article type (e.g., advertisements).

### Analytical method

This research used Multimodal Critical Discourse Analysis (MCDA) to examine both visual and written representations [47]. MCDA is concerned with the ideologies underlying the communicative choices made in texts and images, and how these may reflect or shape the wider social context, particularly power imbalances and the stigmatisation of a social group [41]. In this study, communicative choices refer to written representations (e.g., text) and visual representations (e.g., photographs) that are used within the newspaper articles, hence the ‘multimodal’ focus within CDA, which is also known as Critical Discourse Studies.

Analysis drew upon stigma theory, van Leeuwen’s work on the representation of social actors [48] and the Visual Discourses of Disability (ViDD) framework [49]. A summary of our analytical framework can be found in Table 2. Regarding stigma, the framework is particularly informed by Link and Phelan’s modified labelling theory for stigma [50], as indicated by the following interrelated focuses:

**Table 2:** Summary of analytic framework.

Visual analysis	Written/textual analysis
ViDD: *Perspectivising (focus on disability)* → *Personising (focus on the person) cline* • Distance (long, mid or close shots + focus on disability vs person), • Involvement/detachment (via oblique/frontal angles) • Gaze (indirect/direct) • Inclusion/exclusion in image • Generic/specific social actors • Group homogenisation/group differentiation/individual ViDD: *Enabling* → *Disabling cline* • Affect (positive/negative) • Visual rhetoric (realism/‘supercrip’ narrative/exotic/sentimental) • Viewer power (lower/equal/higher angle) • Exclusion • Role (agent/no action/patient) Additional visual considerations: • Setting • Who is shown (e.g., people with or without HL and D/deafness/ ages/ genders/ ethnicities/ celebrities vs non-celebrities) • Use of colour • Non-person or disability focused images that do not align with the ViDD framework (e.g., buildings, people who do not have HL or D/deafness)	• Foregrounded/backgrounded/excluded discourses of HL and D/deafness • What psychosocial impacts are explored in the articles? • Included/excluded perspectives • Article authors • Quoted individuals/groups • Other individuals/groups mentioned • How are people with HL and D/deafness positioned? • Attributed characteristics (appearance, personality, categories, etc.) • Allocated roles and actions • Individualised vs collective/ generic vs specific • Overall distance/proximity to readers (e.g., do pronouns include readers and people with HL and D/deafness in the same or different social groups?) • Terminology used: does it align with HL and D/deafness guidelines?
**Image and text**	
• How do the images and text reinforce and/or contradict each other? • At the levels of: • image and caption • news article • across the articles

**(1) How people with HL and D/deafness are labelled and (negatively) stereotyped.** This focus underlies much of Table 2 due to our concern with positioning (e.g., what attributes and (in)actions are attributed to people with HL and D/deafness, visually and linguistically?). We were concerned with both identifying stereotypes that occurred across the dataset and in relating these, where possible, to the wider literature. Stereotypes could also be linked to negative attitudes towards HL, for instance, that HL should be hidden [21].
**(2) Whether/how people are socially othered.** This includes consideration of features of visual and textual proximity/distance (e.g., gaze, pronoun use) to help explore whether an ‘us’ vs ‘them’ is established.
**(3) Any examples of status loss, discrimination and power imbalances.** This includes a concern with who writes and is quoted in the articles, the visual exercise of power (e.g., regarding angle) and the roles and actions associated with people with HL and D/deafness. For example, do the articles cover being discriminated against or themselves discriminate against these groups?

For visual analysis, ViDD provided a critical discursive framework that applies van Leeuwen’s foundational work on representation and social actors to the context of visualisations of disability [48,49]. ViDD theorises that there are four types of visual representations that may be used to represent people with disabilities. These four types of visual representation align with the two clines (i.e., the continuums of *perspectivising* → *personising* and *enabling* → *disabling*) detailed in Table 2.

**(1) Empowering (enabling and personising):** focusing on the person as an active individual, undifferentiated from non-disabled people.
**(2) Advocating (enabling, perspectivising):** foregrounding the disability in an inclusive, enabling way.
**(3) Handicapping (disabling, personising):** person presented in a negative or non-participatory way.
**(4) Othering (disabling, perspectivising):** focusing on the disability in a disabling manner that socially ‘others’ the person.

We combined a consideration of the overarching ViDD categories with a focus on specific visual choices within the clines (e.g., gaze and action) and additional considerations (e.g., visual subjects that were not included in the ViDD framework). This provided the necessary flexibility to explore patterns across this specific dataset.

Overall, our analysis is guided by Fairclough’s [51] three-dimensional approach to CDA (of which MCDA is the multimodal branch), which considers the level of (1) the text, (2) discursive practice, and (3) social practice. Therefore, we sought to contextualise our descriptions of specific textual features (e.g., camera angle) in relation to social practice and discursive practice, which considers production, distribution and consumption, here, specifically focusing on article authors and readership. We considered the articles within the wider social context, namely how they might reflect and help shape current norms and social values surrounding HL and D/deafness in the UK.

The analysis was led by the first and second authors, which entailed developing themes in line with the MCDA approach. First, the authors immersed themselves in the data by reading and re-reading the selected newspaper articles. They made extensive notes about the main topics of the articles, points of discursive tension, and the people/perspectives that were foregrounded (and backgrounded or excluded) overall. They coded the text and images from the articles by applying the analytical framework (see Table 2). Rather than constraining visual analysis to the four pre-existing categories established by ViDD, the authors categorised images according to what visual features within the framework (which includes but is not limited to ViDD) were particularly salient across this dataset. For instance, direct gaze was a particularly popular visual strategy for the images in this dataset and within a focus on actions (see ‘role’ in Table 2), a specific subgroup emerged relating to communicating with HL using an interpreter and/or sign language. From this more inductive approach to categorising images, links could then be made to the four overarching ViDD categories when appropriate and helpful for the data. Throughout, attention was paid to the relationship between text and images, notably regarding how they might reinforce and/or contradict each other at the levels of image and caption, news article and across the articles. This approach enabled close analysis of examples from the data to ground the analysis in the visual and linguistic features identified. The first author developed initial themes by identifying patterns in the coded data. These themes were then reviewed and refined in collaboration with the second author.

Recognised strategies for enhancing the rigour of the qualitative analysis were used [52]. First, peer debriefing was used; where the analysis was reviewed by the research team to support or challenge the analysts’ interpretations. Disconfirming evidence analysis also took place, which entails searching for data that conflict with the main findings to ensure any such data are not overlooked [52]. Finally, the research team kept reflexive notes throughout (S3 File).

### Patient and public involvement

A panel of seven PPI contributors with lived experience of HL and D/deafness were consulted throughout. Initial meetings were held with five PPI contributors to obtain feedback on the research objectives and scope. Additional meetings were conducted, where four PPI contributors gave their input on the analysis and made recommendations for future media representations of HL and D/deafness.

## Results

**Fig 1:**
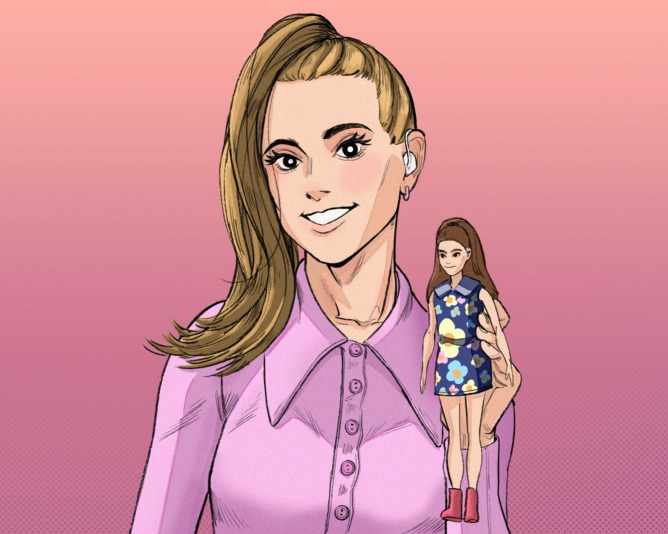
From Article 15, captioned “Incredible: [Celebrity] scored an incredible achievement for the Deaf community as she starred in the first ever hearing impaired Barbie doll campaign earlier this year”.

**Fig 2:**
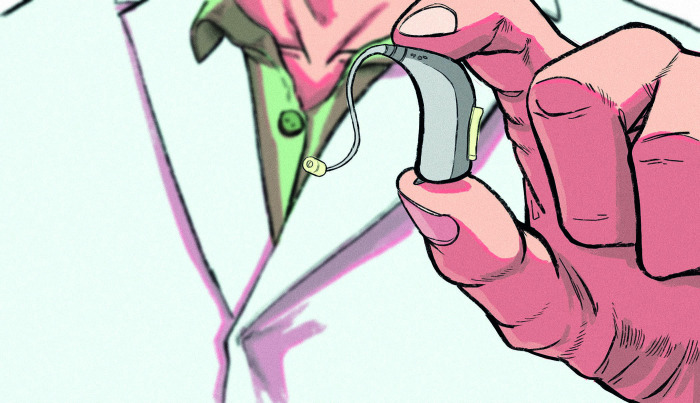
From Article 6, captioned “The latest hearing aids are unobtrusive and help maintain good hearing. Certain types of dementia have been linked to deafness”.

**Fig 3:**
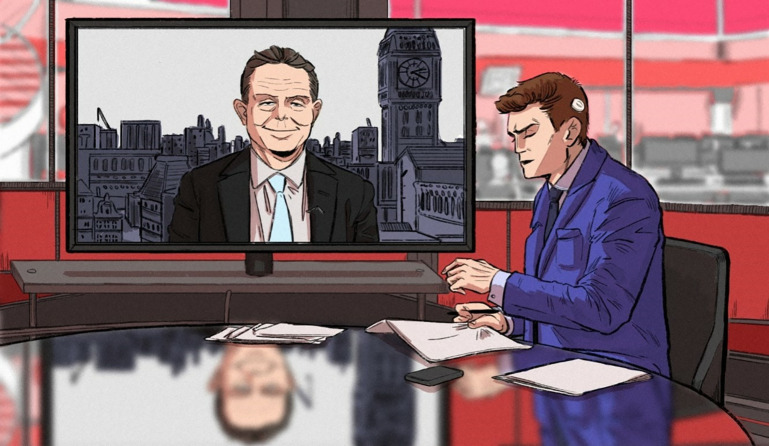
From Article 19, captioned “IN PLAIN SIGHT: [Newsreader] presenting [the] news with his white hearing aid implant clearly visible”.

Across the 20 articles (and 53 images) sampled, there are multiple noteworthy discursive tropes surrounding HL and D/deafness in UK society. This will be discussed in relation to three key themes identified across the articles: (1) representing social progress, (2) missing people and perspectives, and (3) (in)visibility in a “hearing world”. For ease of reference, the newspaper articles have been numbered 1–20 and are referenced by these numbers below (See S4 File for a full list of the articles). Images and text were analysed together to enable consideration of their relationship within articles. In accordance with the MCDA approach, the findings were contextualised through referring to published literature within the field throughout the Results Section. Artist’s impressions of images from the articles have been used for Figs 1–3.

### Theme 1: Representing social progress

The majority of articles report on some form of social progress as their main story. This includes technological advancements, such as a new subtitling app or a haptic suit for concert goers with HL or D/deafness (4,5,9). It also includes first-time events that signal greater inclusion and representation of people with HL or D/deaf people (1,2,7,13,14,16,18). Examples include the first Barbie doll with hearing aids, the first deaf contestant for a popular dating show, and a law change enabling jurors to have sign language interpreters in court. Perpetuating this sense of advancement are metaphors of journeying towards a more inclusive society (“really positive *step towards* inclusion”, 7) and of eliminating barriers while doing so (e.g., “Smashing through barriers”,3; “breaking boundaries”,7; “further break down barriers for our community”,1). As well as indicating a defiance of the restrictive *status quo*, the repeated use of destructive verbs (“breaking”, “smashing”) infers a sense of finality for the dismantling of such barriers, much as smashed objects cannot be re-pieced together. Contributing further to this sense of social progress, Deaf adults reflect on this newfound “mainstream visibility” (3) and contrast it to their past experiences. These reflections usually prompt the conclusion (implicit or explicit) that “my life would have been different if I’d experienced something similar [to the current improvements in representation and inclusion]” (11).

Visually, this social progress discourse is realised through the overwhelming use of images that portray people with HL or D/deaf people as individuals who are active members of society that viewers are encouraged to connect with through various framing choices. Notably, 28 of the total 53 images depict people with HL or D/deaf people looking directly at the camera, which invites viewers to form an imaginary social connection through mimicking real-life eye-contact [53]. Fig 1 offers one example of this visual trope. The friendly nature of this imagined relationship is signalled through recurring uses of a close-up shot. This mimics the closer proximity that tends to be associated with greater social intimacy (i.e., we tend to get physically closer to people we know and like). It is also signalled through the frontal and eye level angle, which is indicative of being part of the same social world, and of being social equals [53]. A further 13 images (Fig 3) show individuals with HL and D/deaf people being engaged, often alongside others, in an activity (e.g., dancing, celebrating, cooking, acting) or in the act of communicating through signing, having an interpreter, or interacting with hearing individuals. Thus, whether engaging with viewers through a direct gaze (e.g., Fig 1) or engaging with other people or in actions (e.g., Fig 2), individuals with HL are depicted as agentive and socially involved individuals. Furthermore, they are often smiling, which is a key signal of positive affect. These features are associated with personising (i.e., focusing on the individual) and enabling (rather than disabling) representations, and as thus being empowering visual tropes in a disability context [49].

Overall, the articles tend to visually and linguistically foreground individual people with HL and individual D/deaf people (exactly who these individuals are and are not will be returned to shortly). As such, photographs tend to be used of the person (or people) being featured (with even Beethoven having a painted portrait; see 4). While HL and D/deafness is the feature overwhelmingly incorporated into the descriptions of individuals, this can be alongside other identity features, as shown with “She looks amazing in a bikini, has a great bubbly personality and just happens to be deaf” (16). The articles in the present study regularly feature the voices and perspectives of individuals with HL and D/deaf people. This facilitates the inclusion of different viewpoints across the articles regarding what it means to experience HL or D/deafness, particularly the latter. Most frequently, HL and D/deafness is categorised as, or otherwise associated with, ‘disability’ (1,3,5,11,12,15). However, other views are also acknowledged, with one deaf celebrity representing being deaf as “not a disability” but “a superpower” (1). This is critiqued by an author from the Deaf community, who argues that most of this community, “want to be treated the same as everybody else, not to be put up on a pedestal as ‘superhuman’ or…‘inspiration porn’” (1). This critique aligns with criticisms of the ‘supercrip’ narrative, whereby people with disabilities can be overly glorified in media [34,35]. Simultaneously, someone can “love being deaf” but find it “exhausting sometimes”, in part through being a “lone voice” and thus being “forced to ‘be political without wanting to be political’” when making the workplace “accessible for deaf [people]” (15). The pathological model of deafness is also referenced as influencing individuals’ self-concept as having a “physical ‘deficit’” (8), or as its converse, of celebrating Deaf identity and culture (3) [31,54].

Specifically, four articles (20%) are authored by someone who explicitly identifies as D/deaf (1,3,5,8), while thirteen directly quote people with HL and D/deaf people, who are by far the most frequently used source (1,2,4,5,7,11,12,13,14,15,16,19,20). Overall, then, 75 per cent of articles directly feature the voice of someone with HL or D/deafness, although there is a large variation regarding exactly how much of the person’s perspective is featured. Media communication guidelines, produced by organisations such as the National Association of the Deaf [55], advise the media to consistently include the first-hand viewpoints of d/Deaf people regardless of the story’s angle (e.g., medical, legal, social, cultural, etc.). In addition, they advise carefully considering relevant sources to best reflect the diversity of this community. While a 75 per cent inclusion rate signals progress towards inclusion, then, it is also worth noting that this remains very similar to the 71 per cent inclusion rate of the voices of D/deaf people observed by Haller [56] in American news coverage between 1986–1990.

### Theme 2: Missing people and perspectives

Whilst there are advantages to the above emphasis on the individual within a social progress discourse, it is also arguably problematic in two main ways. Firstly, certain types of people and experiences are foregrounded, while others are excluded. Secondly, such individualisation risks backgrounding issues of systemic change. First, the individuals featured tend to be celebrities, namely TV personalities and successful writers (for exceptions, see 5, 8, 14, 18). As social elites, celebrities are considered especially newsworthy [57], and in the articles, they are often positioned as “an inspirational figure” who can change “perceptions of what it means to be deaf” (12). Yet, an overemphasis on people with exceptional achievements or status is unattainable for most people, and risks backgrounding more everyday experiences and barriers associated with HL and D/deafness. Moreover, all featured individuals are of working age (approximately aged 20–45). Whilst this challenges the stereotype that people with HL are always older adults [58], it also perpetuates the widespread backgrounding, and even exclusion, of older people (including those with HL or D/deaf people) in popular media [29,31,59]. Relatedly, most articles appear to foreground white, cisgendered people, further perpetuating a lack of diversity in representations of people with HL or D/deaf people [29].

Furthermore, focusing on how individuals engage with and embody social progress risks backgrounding broader systemic change (for the exception, see article 14, which reports on a new law that gives D/deaf jurors access to interpreters). Notably, multiple moments of social progress (such as a televised sign language story for children) are tied to a particular campaign (11,12) or to Deaf Awareness Week (2,11,20). The temporary nature of these moments of progress leads to the question of whether they are worth reporting *because* they are and will continue to be rare. It appears that these moments do not reflect a comprehensive and sustainable inclusion of people with HL and D/deaf people within larger social structures, as the irrevocability of the metaphor of “smashing” or “breaking” barriers might suggest. Recognising this tension, one Deaf and disabled journalist notes that, as well as a TV dating show including their first deaf contestant, the TV itself must be accessible through subtitles, which the show’s broadcaster previously failed to do (1). Elsewhere, an app that converts speech into subtitles and translates different languages is presented as “truly life-changing”. However, it only offers a free user option for English, with non-English speakers needing to pay £19.99 per month, and the top-tier costing £49.99 a month, a disparity that remains unmentioned in the article (9).

Moreover, although multiple articles report on communication-oriented campaigns, there remain examples of outdated, inaccurate or ambiguous representations of HL in the dataset. This indicates a need for further education and guidance on HL and D/deafness for media institutions themselves. For instance, the term “hearing impaired” is occasionally used (7,17), which has been criticised as outdated and inaccurate for making assumptions about a person’s abilities, rather than just describing the person [55]. In terms of hearing technologies, despite its caption discussing “the latest hearing aids”, the image shown in Fig 2 depicts a somewhat outdated hearing aid model. Article 16 uses the terms hearing aids and cochlear implants interchangeably to describe an individual’s hearing device, conflating the two, despite the images clearly depicting a cochlear implant. Such examples are important to address, since media institutions contribute to the misconceptions of HL, D/deafness and hearing technology that have been identified amongst the public [60].

### Theme 3: (In)visibility in a “hearing world”

Having considered who and what is (and is not) made visible in these articles, the very concept of (in)visibility will be examined further, specifically as it pertains to experiencing HL and D/deafness in a primarily “hearing world” (2,3). This world is shown to underrepresent, misrepresent, undermine and exclude people with HL or D/deaf people (3,8,11,12). Accompanying the discourse of social progress, then, is one of stigma and social barriers in a hearing-oriented society. A common thread throughout the accounts of people with HL or D/deaf people and articles more broadly is an anxiety about *showing* HL or D/deafness. This can be linked to “ableism, first systemic, then internalised” (3), and the resulting “stigma” surrounding “wearing a hearing aid” (6) and “intense shame about my deafness” (11). Internalised stigma, or shame, is a key psychosocial consequence of HL/deafness in these articles, alongside a sense of isolation (3,4,9,12,19), mental health difficulties, including psychosis, suicidal ideation, and depression (1,4,6,8,9,12,15). Indeed, while being associated with making social progress by “helping break down […] barriers” (12), that the articles report on multiple campaigns tackling the psychosocial impact of HL and D/deafness. This highlights the many social barriers that such individuals *still* face in a hearing-oriented world, which is further reinforced by campaign statistics, including that 50 per cent of D/deaf survey respondents feel isolated and frustrated (12).

Reflecting (and arguably reproducing) the stigma surrounding HL and D/deafness, multiple articles foreground the (in)visibility of hearing aids and cochlear implants. Assuming a preference to “hide” such signs of HL and deafness (8,11), one article claims that the “latest hearing aids are unobtrusive” (6). Only seven of the 41 images of people with HL and deafness clearly show a hearing aid or cochlear implant (e.g., Figs 1 and 3). This could be interpreted as a positive move away from defining people solely in relation to this identity feature, or as perpetuating the normalisation of hiding HL or deafness. The newsworthiness of making HL or deafness visible is exemplified by Fig 3, which foregrounds that a newsreader’s “white hearing aid implant [is] *clearly visible”* and is “*in plain sight*”. The newsreader is quoted explaining that “I wanted it to stand out, to make sure people with hearing aids know they’re not alone”. Here, visibility is associated with building a sense of community within an often-isolating hearing-oriented world. Elsewhere, a celebrity is “praised for her bravery in showing off her [cochlear implant]” in a modelling campaign. She positions herself as “inspirational because of my hearing and my cochlear implant. I inspire quite a lot of people because I’m really open about it” (16). To ‘show off’ a cochlear implant situates this choice as a piece of activism, and perhaps a fashion statement, rather than as a device that supports her communication and is a normal part of daily life. While not intending to diminish the difficulties that people with HL and D/deafness face, evaluating celebrities as being “brave” and “inspirational” for wearing a cochlear implant risks being interpreted as patronising [55] or as perpetuating the ‘supercrip’ narrative. Indeed, many members of the Deaf community would contest the notion that they have a deficit that needs to be ‘fixed’ or ‘cured’ [61]. Similarly, Fig 1 positions a celebrity’s involvement with the first Barbie doll with hearing aids as “an incredible achievement”. Alternative framings are ignored, including that this is a necessary and long overdue step towards better representation, and that companies should be held accountable to become more inclusive. Explicitly engaging with this issue, Article 6 claims that society should “normalise the wearing of a hearing aid and the stigma will then be reduced”.

## Discussion

This study explored the representation of HL and D/deafness in the UK news media through taking an MCDA approach to a case study of 20 news articles. Three key themes were identified. The first concerned the representation of social progress, including greater inclusion of people with HL or D/deaf people. The second discussed the lack of diverse narratives and perspectives, including scant representations of older adults. The third concerned the stigma and social barriers associated with living in a hearing-orientated world, including tensions regarding making HL (in)visible. Combined, the analysis suggests a conflict between the news’ foregrounding of social progress regarding more inclusive practices (both in the media and in everyday life) and the perpetuation of stigma.

Across the twenty articles specifically featuring HL and D/deafness, accounts from a person with HL or D/deaf people occurred 75 per cent of the time. Within this, 20 per cent of all articles were authored by a journalist with HL or D/deafness. In many ways, this aligns with best practice guidelines for the media that promote foregrounding the perspectives of people experiencing HL and D/deafness [55]. It also aligns with stigma theorists’ recommendation to challenge stigma through promoting contact between people who are stigmatised and the majority group [62]. However, the intersectionality of stigma with other social attributes (e.g., age, socioeconomic status, ethnicity and sexual identity) is also apparent in whose perspectives are foregrounded in these articles, and whose are backgrounded, or even absent.

The prominence of celebrity voices in this dataset is perhaps unsurprising considering the newsworthiness of figures considered to be social elites [57], and their high social status can be beneficial for anti-stigma initiatives [63]. Arguably, celebrities’ positions of power can support their influence as advocates and as role models (here, showcasing a range of successes, including as authors and presenters). They can also help to normalise experiencing HL and D/deafness in a hearing-dominated world, as indeed multiple individuals indicate in this dataset, such as the discussion of celebrities purposely showing their hearing devices. Through focusing on such figures and their achievements, and through the predominance of images that display positive affect and social engagement for people with HL or D/deaf people, the articles challenge previous misrepresentations and misconceptions of this population. These include misconceptions of being more isolated, incapable, dehumanised, emotionally negative and even ‘defective physically, emotionally, cognitively, socially, and psychologically’ than the rest of the population [31,55,64]. Against this background, it is encouraging that the images in this dataset tend to align with personising and enabling representations, and thus with empowering visual tropes in a disability context [49].

However, it is worrying that the celebrities and other featured individuals with HL or D/deaf people are (as far as can be discerned) overwhelmingly white, cisgender and working age (approximately aged 20–45). This reiterates existing findings in other cultural contexts [e.g., 29,31,59]. The emphasis on working-age people with HL or who are D/deaf may be interpreted as constructive, by challenging the stereotype that HL just affects older adults [58,65]. Young people with HL and D/deaf young people are a relatively small group but they can experience significant challenges in terms of education, employment, relationships and mental health [66,67]. This may differ somewhat to the consequences that older people experience, especially if they develop HL in later life. However, the *extent* of the absence of older people in the images and text could be concerning, given that the majority of people living with HL are older adults. This reiterates the underrepresentation of older people in both the media more broadly, which can be tied to ageism, specifically, the stigma of older age [59,68].

Arguably, the articles’ emphasis on social progress is also undermined by backgrounding issues of systemic change and inadvertently reinforcing narrow stereotypes, and even stigma, through their portrayals of people with HL and D/deaf people. For example, multiple articles promote what may be referred to as “inspiration porn” (1) or the ‘supercrip’ narrative, including through describing individuals as inspirational due to clearly showing their hearing devices [34,35]. That such actions are newsworthy, let alone inspirational, implicitly signals that *HL is expected to be hidden*, and thus wearing a hearing device publicly becomes heroic, rather than normalised. Indeed, the article that foregrounds hearing devices being “unobtrusive” (rather than particularly effective for someone with HL or D/deaf people) aligns with previous observations that the media emphasises the small size of hearing aids. This can increase the stigma surrounding HL and hearing aids by reinforcing that they should be hidden [21,58]. Notably, the frequent positioning of individuals with HL or D/deafness as inspirational role models and political advocates, while certainly challenging prior stereotypes of dependency and isolation, is itself also a restrictive stereotype. This separates people with HL and D/deaf people and places undue responsibility on individuals to drive social change, which can cause harm. Throughout, steps to include people experiencing HL and D/deafness (e.g., the first Barbie doll with hearing aids) are recurringly positioned as positive without questioning why some of these moves are only happening now. A quarter of the articles are in response to specific campaigns or to Deaf Awareness Week, despite the year-long data collection window. It is important to question whether the reported moments of inclusion are newsworthy because they are and will likely *continue* to be rare, rather than reflecting a comprehensive and sustainable inclusion of people with HL and D/deaf people. Similarly, considering that these articles were selected because of their focus on HL and D/deafness, it is important to ask how articles without this explicit focus represent people experiencing HL and D/deafness and their views. Equally, missing from this dataset’s representation of hearing technologies is the rejection of cochlear implants by some members of the Deaf community, as they may pose a threat to Deaf culture [61]. The coverage of diverse opinions both within the Deaf community and amongst people experiencing HL and deafness is an important focus for future research.

Based on the analysis and PPI meetings, the following recommendations for how HL should be represented in the media were developed. HL representations (both visual and textual) should be more diverse, including through representing people with a range of social identities (regarding age, ethnicity, etc.). They should also include a range of stories (e.g., positive/negative, ordinary, factual), rather than, for instance, only inspirational stories. Such representations should be led, as much as is possible, by people who themselves have diverse experiences and perspectives of HL and D/deafness. Indeed, the value of this is indicated by the more multifaceted compilation of views on HL and D/deafness and its representations that were expressed by interviewees and journalists experiencing HL and D/deafness in these articles. A focus on individuals and their experiences should not be at the expense of scrutinising the roles and responsibilities of broader social structures and organisations, such as governments, healthcare services and corporations. Overall, the news should raise sustained awareness of HL and D/deafness, including more informative articles about different types of HL and the experience of being D/deaf. More informative and nuanced articles could reduce the misconceptions the public may have about HL and D/deafness [60]. Within this, it is important to inform readers about (mental) health in relation to HL and D/deafness to help educate the public, help people to feel less alone and encourage individuals to seek support. The media play a significant role in determining society’s perceptions of health conditions. Thus, developing a better understanding of and guiding media representations has the potential to improve the management of conditions such as HL by highlighting (mis)perceptions that need to be addressed [30]. The authors hope that this and future research can aid public communication about HL and inform best practice recommendations, especially for those working in public health, clinical research, clinical practice, charities, and the media to help destigmatise HL and D/deafness.

A key strength of this research was the contribution of the PPI panel, which ensured that the voices of individuals with HL were reflected in the research aims, data analysis, and recommendations. One limitation of this study is its small sample of newspaper articles from 2022–2023, which cannot be expected to capture all representations of HL and D/deafness, despite having reached data saturation during screening. Yet, the goal of MCDA is to provide a comprehensive exploration of how a phenomenon might be portrayed within a particular context and the social implications of this, as opposed to providing an account of *all* possible representations. This paper aims to show how MCDA, an established approach for critically engaging with written and visual messages, here regarding health conditions, can be usefully applied to a HL and D/deafness context [69]. In doing so, this paper contributes further insights to relevant academic fields, particularly discourse and disability studies, through attending to multimodal representations in an under-explored and complex context: HL and D/deafness. Further research is needed, including the application of MCDA to other prominent sources of information about HL and D/deafness, such as television or social media, and to newspaper coverage in other countries. Future research should also include partnering with people living with HL and D/deaf people to co-produce alternative representations to those critiqued here and elsewhere. It is important to critically discuss this issue to reduce misconceptions and to guide the media and those who communicate with the public about HL and D/deafness.

## Supporting information

S1 File Standards for Reporting Qualitative Research (SRQR) Checklist. (DOCX)

S2 File Nexis database search string. (DOCX)

S3 File Reflexivity. (DOCX)

S4 File Table of newspaper articles included in the analysis. (DOCX)
