# Supplementary material for: “Smashing through barriers”? A multimodal critical discourse analysis of media representations of hearing loss and D/deafness
Source: PLoS One. 2026 Feb 13;21(2):e0342462. doi: 10.1371/journal.pone.0342462 (PMC12904403; doi:10.1371/journal.pone.0342462)
Supplement: S1 File — (DOCX) [file pone.0342462.s001.docx]

**S1 File**

**Standards for Reporting Qualitative Research (SRQR) Checklist.**

| **Title and abstract** | | **Sections** |
| --- | --- | --- |
|  | **Title**  Concise description of the nature and topic of the study. Identifying the study as qualitative or indicating the approach or data collection methods (e.g., interview, focus group) is recommended. | Title Page |
|  | **Abstract**  Summary of key elements of the study using the abstract format of the intended publication; typically includes background, purpose, methods, results, and conclusions. | Abstract |
|  |  |  |
| **Introduction** | |  |
|  | **Problem formulation**  Description and significance of the problem/phenomenon studied; review of relevant theory and empirical work; problem statement. | Introduction |
|  | **Purpose or research question**  Purpose of the study and specific objectives or questions. | Introduction |
|  |  |  |
| **Methods** | |  |
|  | **Qualitative approach and research paradigm**  Qualitative approach and guiding theory if appropriate; identifying the research paradigm is also recommended. | Introduction, Materials and methods |
|  | **Researcher characteristics and reflexivity**  Researchers’ characteristics that may influence the research, including personal attributes, qualifications/experience, relationship with participants, assumptions, and/or presuppositions; potential or actual interaction between researchers’ characteristics and the research questions, approach, methods, results, and/or transferability. | S3 File |
|  | **Context**  Setting/site and salient contextual factors. | Materials and methods |
|  | **Sampling strategy**  How and why research participants, documents, or events were selected; criteria for deciding when no further sampling was necessary (e.g., sampling saturation). | Materials and methods |
|  | **Ethical issues pertaining to human subjects**  Documentation of approval by an appropriate ethics review board and participant consent, or explanation for lack thereof; other confidentiality and data security issues. | Materials and methods |
|  | **Data collection methods**  Types of data collected; details of data collection procedures including (as appropriate) start and stop dates of data collection and analysis, iterative process, triangulation of sources/methods, and modification of procedures in response to evolving study findings. | Materials and methods |
|  | **Data collection instruments and technologies**  Description of instruments (e.g., interview guides, questionnaires) and devices (e.g., audio recorders) used for data collection; if/how the instrument(s) changed over the course of the study. | Materials and methods |
|  | **Units of study**  Number and relevant characteristics of participants, documents, or events included in the study; level of participation. | Materials and methods, Table 1 |
|  | **Data processing**  Methods for processing data prior to and during analysis, including transcription, data entry, data management and security, verification of data integrity, data coding, and anonymization/de-identification of excerpts. | Materials and methods |
|  | **Data analysis**  Process by which inferences, themes, etc., were identified and developed, including the researchers involved in data analysis. | Materials and methods, Table 2 |
|  | **Techniques to enhance trustworthiness**  Techniques to enhance trustworthiness and credibility of data analysis (e.g., triangulation). | Materials and methods |
|  |  |  |
| **Results/findings** | |  |
|  | **Synthesis and interpretation**  Main findings (e.g., interpretations, inferences, and themes); might include development of a model, or integration with prior research or theory. | Results |
|  | **Links to empirical data**  Evidence (e.g., quotes, field notes, text excerpts, photographs) to substantiate analytic findings. | Results, Figures 1-3 |
|  |  |  |
| **Discussion** | |  |
|  | **Integration with prior work, implications, transferability, and contribution(s) to the field**  Short summary of main findings; explanation of how findings and conclusions connect to, support, elaborate on, or challenge conclusions of earlier scholarship; discussion of scope of application/generalisability; identification of unique contribution(s) to scholarship in a discipline or field. | Results and Discussion |
|  | **Limitations**  Trustworthiness and limitations of findings. | Discussion |
|  |  |  |
| **Other** | |  |
|  | **Conflicts of interest**  Potential sources of influence or perceived influence on study conduct and conclusions; how these were managed. | Title Page |
|  | **Funding**  Sources of funding and other support; role of funders in data collection, interpretation, and reporting. | Title Page |
|  |  |  |

**Reference: O’Brien B, Harris I, Beckman T, Reed D and Cook D** (2014) Standards for reporting qualitative research: A synthesis of recommendations. *Academic Medicine: Journal of the Association of American Medical Colleges* 89(9), 1245–1251.
