# Supplementary material for: “Smashing through barriers”? A multimodal critical discourse analysis of media representations of hearing loss and D/deafness
Source: PLoS One. 2026 Feb 13;21(2):e0342462. doi: 10.1371/journal.pone.0342462 (PMC12904403; doi:10.1371/journal.pone.0342462)
Supplement: S2 File — (DOCX) [file pone.0342462.s002.docx]

**S2 File**

Nexis database search string:

| ‘hearing loss’ OR ‘Deaf’ OR ‘Deafness’ OR ‘hard of hearing’ OR ‘hearing difficulty’ OR ‘hearing impaired’ OR ‘hearing impairment’ OR ‘hearing aid’ OR ‘cochlear implant’ OR ‘sign language’ OR ‘BSL’. |
| --- |

*Note: ‘hearing aid’ and ‘cochlear implant’ also returns results for ‘Hearing aids’ and ‘cochlear implants’.*
