# Supplementary material for: “Smashing through barriers”? A multimodal critical discourse analysis of media representations of hearing loss and D/deafness
Source: PLoS One. 2026 Feb 13;21(2):e0342462. doi: 10.1371/journal.pone.0342462 (PMC12904403; doi:10.1371/journal.pone.0342462)
Supplement: S3 File — (DOCX) [file pone.0342462.s003.docx]

### **S3 File**

### **Reflexivity**

It is important for researchers to disclose their social positioning and potential biases which inevitably influence analysis of the data (Creswell & Miller, 2000). Notably, the first author carried out this research as an MSc: Mental Health student in a UK university. She has a psychology background and is learning British Sign Language, which means that she had a pre-existing interest in this field. The majority of her co-authors also have a background in psychology and are based in a hearing sciences department within that university. They have extensive experience of investigating age-related hearing loss using qualitative and quantitative methods. They had a particular interest in the accuracy of the descriptions of hearing loss and audiological interventions within the articles, as well as portrayals of the association between hearing loss and mental health and stigma. Another co-author specialises in dementia research at another UK university, with a background in linguistics and multimodal critical discourse analysis, but little prior knowledge of hearing loss. Another co-author is an academic old age psychiatrist with research interests in dementia and hearing loss. He has moderate hearing loss and wears hearing aids. It is important to note that there are always multiple possible interpretations of the same texts (Machin & Mayr, 2023), and so other researchers or individuals with hearing loss may offer different findings to this interpretation. The majority of the research team do not have hearing loss and thus could be regarded as ‘outsider’ researchers. Therefore, it was crucial to consult a Patient and Public Involvement (PPI) panel to obtain the perspective of ‘insiders’ (i.e., people with lived experience of hearing loss or D/deafness).

**References:**

**Creswell J and Miller D** (2000) Determining Validity in Qualitative Inquiry. *Theory Into Practice* **39**(3), 124–130.

**Machin D and Mayr A** (2023) How to Do Critical Discourse Analysis: A Multimodal Introduction. SAGE.
