# Supplementary material for: “Smashing through barriers”? A multimodal critical discourse analysis of media representations of hearing loss and D/deafness
Source: PLoS One. 2026 Feb 13;21(2):e0342462. doi: 10.1371/journal.pone.0342462 (PMC12904403; doi:10.1371/journal.pone.0342462)
Supplement: S4 File — (DOCX) [file pone.0342462.s004.docx]

## **S4 File**

## Table of Newspaper Articles included in the Analysis

| **No.** | **Headline and URL** | **Date** | **Author** | **Newspaper** | **Category** |
| --- | --- | --- | --- | --- | --- |
| 1 | Voices: Love Island has its first deaf contestant – but has it learnt from past mistakes?  <https://www.independent.co.uk/voices/tasha-ghouri-itv-love-island-2022-deaf-b2095266.html> | 06/06/2022 | Liam O’Dell | The Independent | Broadsheet |
| 2 | Rose Ayling-Ellis to sign Cbeebies Bedtime Story  <https://www.independent.co.uk/arts-entertainment/tv/news/rose-ayling-ellis-cbeebies-bedtime-story-b2072719.html> | 06/05/2022 | Connie Evans | The Independent | Broadsheet |
| 3 | The vehicle for my feelings: how sign language helped a deaf author find her voice  <https://www.theguardian.com/books/2022/may/13/the-vehicle-for-my-feelings-how-sign-language-helped-a-deaf-author-find-her-voice> | 13/05/2022 | Sara Novic | The Guardian | Broadsheet |
| 4 | DNA from Beethoven’s hair could explain his deafness  <https://www.thetimes.co.uk/article/dna-from-beethovens-hair-could-explain-his-deafness-h7zm97bj3> | 22/03/2022 | Rhys Blakely | The Times | Broadsheet |
| 5 | GOOD VIBRATIONS: Can a vibrating haptic suit help deaf and hard-of hearing people  <https://www.independent.co.uk/arts-entertainment/music/features/deaf-live-music-concert-vodafone-b2098260.html> | 11/06/2022 | Liam O’Dell | The Independent | Broadsheet |
| 6 | Having a hearing test in your 30s to beat dementia  <https://www.thetimes.co.uk/article/hearing-test-your-30s-beat-dementia-gnt0bz80z#:~:text=People%20in%20their%20thirties%20should,of%20certain%20types%20of%20dementia> | 18/01/2023 | Kat Lay | The Times | Broadsheet |
| 7 | Strictly star Rose Ayling-Ellis inspires first Barbie doll with hearing aids  <https://www.independent.co.uk/life-style/rose-ayling-ellis-barbie-hearing-aids-b2145837.html> | 16/08/2022 | Maanya Sachdeva | The Independent | Broadsheet |
| 8 | I finally decided to wear my hearing loss rather than hide it – I was just exhausted  <https://www.theguardian.com/commentisfree/2022/jul/24/i-finally-decided-to-wear-my-hearing-loss-rather-than-hide-it-i-was-just-exhausted> | 23/07/2022 | Nick Button | The Guardian | Broadsheet |
| 9 | App to put subtitles on real-time conversations goes live  <https://www.independent.co.uk/tech/google-play-store-mandarin-english-italian-french-b2226813.html> | 17/11/2022 | Martyn Landi | The Independent | Broadsheet |
| 10 | Take tests to ensure hearing loss diagnosed early, public urged  <https://www.independent.co.uk/news/health/hearing-loss-test-loneliness-depression-b2257493.html> | 06/01/2023 | Joe Sommerlad | The Independent | Broadsheet |
| 11 | Award-winning poet Raymond Antrobus spent years trying to hide his deafness at work  <https://www.mirror.co.uk/news/health/award-winning-poet-raymond-antrobus-29847226> | 29/04/2023 | Adrian Monti | Mirror | Tabloid |
| 12 | Make the connection Quarter of Brits would struggle to interact with someone who is deaf  <https://www.independent.co.uk/news/uk/home-news/deaf-lip-reading-sign-language-b2161881.html> | 09/09/2022 | Terri-Ann William | The Sun | Tabloid |
| 13 | This Morning fans beg for more after ITV show features it's first ever deaf chef  <https://www.mirror.co.uk/tv/tv-news/morning-fans-beg-more-after-28169232> | 06/10/2022 | Jasmine Allday | Mirror | Tabloid |
| 14 | Sign Language is used in court as deaf jurors with their own interpreters are used for the first time in England  <https://www.dailymail.co.uk/news/article-11260733/Sign-language-used-court-deaf-jurors-interpreters-used-time-England.html> | 29/09/2022 | David Barrett | The Mail | Tabloid |
| 15 | 'I love being deaf': Rose Ayling-Ellis reveals her disability has given her a purpose - but says the responsibility of representing others can be a burden  <https://www.dailymail.co.uk/tvshowbiz/article-11528955/Rose-Ayling-Ellis-says-deaf-given-purpose-feel-like-burden.html> | 12/12/2022 | Geraint Llewellyn | The Mail | Tabloid |
| 16 | Strictly friends, Inside Love Island star Tasha's secret friendship with Eastenders and Strictly star  <https://www.thesun.co.uk/tv/18735040/love-island-deaf-star-tasha-ghouri-celebrity-friend/> | 30/05/2022 | Joanne Kavanagh | The Sun | Tabloid |
| 17 | Chris martin reveals how Dakota Johnson helped make coldplay shows fun for deaf audience  <https://www.mirror.co.uk/3am/celebrity-news/chris-martin-reveals-how-dakota-27419730> | 07/07/2022 | Scarlett O’Toole | The Mirror | Tabloid |
| 18 | Deaf British Airways worker breaks new ground by following in his father's footsteps  <https://www.mirror.co.uk/travel/news/deaf-british-airways-worker-breaks-29796790> | 27/04/2023 | Marjorie Yue | The Mirror | Tabloid |
| 19 | Excruciating moment my hearing aid died as I read the news on television; Lewis Vaughan Jones speaks candidly about the challenge of carrying on his TV career after becoming deaf at 37  <https://www.dailymail.co.uk/health/article-11524007/BBC-newsreader-Lewis-Vaughan-Jones-reveals-moment-hearing-aid-died-read-news.html> | 03/01/2023 | Ethan Ennals | The Mail | Tabloid |
| 20 | Strictly's Rose Ayling-Ellis announces exciting new TV job two years after winning show  <https://www.mirror.co.uk/tv/tv-news/strictlys-rose-ayling-ellis-announces-29810834> | 25/04/2023 | Mia O’Hare | The Mirror | Tabloid |
